# Supplementary material for: Enhancing the energy level of regional digital innovation ecosystems: A configuration perspective
Source: PLoS One. 2024 Oct 1;19(10):e0309229. doi: 10.1371/journal.pone.0309229 (PMC11444408; doi:10.1371/journal.pone.0309229)
Supplement: Attachment — Submitted filename: PLOS ONE.docx [file pone.0309229.s001.docx]

**Reviewer's Report**

The paper titled "Enhancing the Energy Level of Regional Digital Innovation Ecosystems: A Configuration Perspective" explores the mechanisms of "energy level" in regional digital innovation ecosystems. This study, by integrating the "architects" theory, Necessary Condition Analysis (NCA), and Fuzzy-set Qualitative Comparative Analysis (fsQCA), examines the impact of different types of architects and digital innovation habitats on the energy levels of these ecosystems across 30 provinces in China. The main findings indicate that while no single antecedent condition can significantly affect the energy level alone, core innovation actors and digital infrastructures emerge as key factors. The paper identifies multiple concurrent causal relationships among architects and outlines three core configuration paths that drive high energy levels, classifying them as "core actor-infrastructure" driven. The article has its innovative aspects, but it still has shortcomings:

First, the article provides a review and introduction to "regional innovation ecosystems." However, the term changes from "regional innovation ecosystems" to "regional digital innovation ecosystems" lacks clarity. The authors also do not explain what "regional digital innovation ecosystems" are or how they differ from "regional innovation ecosystems." This part should be clearly introduced before Figure 1.

Second, rather than calling Figure 2 a theoretical model, it is more accurately described as the analytical framework of this article, based on the theoretical foundations in Section 2. I believe it should be placed in Section 3 instead of Section 2.

Third, the section on data collection and indicator selection needs to be more detailed and specific. For example, the statement "data from 2018-2020 is chosen for the antecedent conditions" is vague. Specifically, which data from which year was used?

Fourth, the author raised many questions in line 82 that need to be addressed in the conclusion section.

Finally, although the author spends much space introducing and explaining the "Architects" Theory, I do not understand what distinguishes it from the general actors in an innovation ecosystem, or what additional value using the "Architects" Theory provides.

**Additional Points:**

On page four, the author's mention that "Concurrently, the advent of digital technology has revolutionized traditional economic models, giving rise to innovation actors" might confuse the reader. It is unclear whether the term "giving rise" refers to an increase in the number, type, or capabilities of innovation actors, and why.

In line 89, the statement "Review of the literature reveals that research on the development paths of regional innovation ecosystems generally progresses through three stages" could be ambiguous. It might lead some readers to think it refers to the development of regional innovation ecosystems themselves rather than the evolution of research on them, which is often divided into three stages.

At the end of line 106, it feels necessary to clarify which approach the paper uses.

Any references to literature that are in Chinese should have "(in Chinese)" added after them to indicate the language of the sources.
